# Vitamin D Deficiency Predicts Poor Clinical Outcomes in Heart Failure Patients Undergoing Cardiac Resynchronization Therapy

**DOI:** 10.1155/2019/4145821

**Published:** 2019-10-13

**Authors:** P. Perge, A. M. Boros, L. Gellér, I. Osztheimer, Sz Szilágyi, T. Tahin, A. Apor, K. V. Nagy, E. Zima, L. Molnár, B. Merkely, G. Széplaki

**Affiliations:** Heart and Vascular Center, Semmelweis University, Városmajor utca 68, Budapest 1122, Hungary

## Abstract

**Background and Aims:**

Resynchronization therapy (CRT) improves mortality and induces reverse remodeling in heart failure (HF) patients with reduced ejection fraction and wide QRS. Nonetheless, some patients do not improve despite the optimal medical therapy and right indications for device implantation. Therefore, finding biomarkers suitable for identification of those patients is crucial. Vitamin D plays a classic hormonal role in the regulation of bone metabolism and also has physiological functions in wide range of nonskeletal tissues. Based on recent studies, low levels of vitamin D seem to directly contribute to pathogenesis and worsening of HF. We planned to assess the role of vitamin D levels on clinical outcomes of HF patients undergoing CRT.

**Methods and Results:**

We enrolled 136 HF patients undergoing CRT. Total plasma vitamin D levels were measured at baseline and 6 months later. Primary endpoint was 5-year all-cause mortality; secondary endpoint was lack of good clinical response, defined as less than 15% increase of left ventricular ejection fraction after six months. During follow-up, 58 patients reached the primary, and 45 patients reached the secondary endpoint. Vitamin D levels less than 24.13 ng/mL predicted 5-year mortality (*p* = 0.045) and poor clinical response (*p* = 0.03) after adjusting to all significant baseline predictors.

**Conclusion:**

Our study showed that vitamin D deficiency has a significant impact in heart failure patients; it is an independent predictor of lack of midterm clinical response and long-term mortality in patients undergoing CRT. Therefore, monitoring vitamin D status of heart failure patients could be of clinical significance.

## 1. Introduction

Heart failure (HF) bears a major public health impact with constantly growing incidence, despite continuous improvements in prevention, diagnosis, and therapy [1]. Cardiac resynchronization therapy (CRT) is an effective therapeutic option for symptomatic HF patients with severely reduced left ventricular ejection fraction (LVEF) and wide QRS. In most patients, mortality and morbidity are reduced, while functional capacity and HF symptoms are improved [2, 3]. Despite the repeated refinements in the guidelines for optimal patient selection, poor clinical response to CRT is still prevalent [4], thus recognizing further predictors of outcome is crucial.

Vitamin D, initially known as a key hormone of bone metabolism, has several extraskeletal physiologic functions. Based upon recent studies, vitamin D is an important regulator of the renin-angiotensin-aldosterone system (RAAS), inflammatory cytokines, and extracellular matrix (ECM) turnover. Moreover, vitamin D deficiency directly contributes to pathogenesis of HF by the loss of above modulating mechanisms, causing remodeling of the heart [5]. Numerous cross-sectional and longitudinal studies showed that vitamin D deficiency is associated with increased risk of HF; in addition, worse prognosis of already diagnosed HF was also demonstrated [6]. Interestingly, vitamin D supplementation in primary or secondary prevention of HF is controversial; there is no definitive evidence supporting a favorable role of vitamin D supplementation [7].

There is limited data in the literature assessing the role of vitamin D deficiency in predicting clinical response to CRT. The results of previous small-scale studies suggest that patients with low levels of vitamin D show inadequate six-month clinical response to CRT [8, 9]; however, no data exists regarding hard endpoints. The aim of our study was to determine the predictive value of vitamin D deficiency on the long-term mortality after CRT and confirm the association with poor midterm clinical response.

## 2. Methods

### 2.1. Study Population

141 consecutive HF patients were enrolled to our prospective, single-center, observational study. The purpose of the study was to evaluate the prognostic value of various biomarkers in a cohort of HF patients previously described in details, including routine laboratory markers, uric acid, complement components, and novel HF biomarkers [10–14]. This present study focused on the role of vitamin D levels in the prognosis after CRT implantation.

We enrolled chronic heart failure patients with optimal medical therapy, symptomatic HF (New York Heart Association functional class II-IVa), left ventricular ejection fraction (LVEF) below 35%, and wide QRS complex (>120 msec) in the baseline electrocardiogram (ECG). Vitamin D supplementation was not included in the medical therapy before enrolment and during the follow-up. The patients underwent CRT implantation according to the current guidelines [15] in the Heart and Vascular Center of Semmelweis University, Budapest, between September 2009 and December 2010. Severe systemic inflammatory and hematologic diseases and active malignancies were considered as exclusion criteria; we excluded 4 patients based on these conditions; furthermore, we did not have complete dataset of one patient.

### 2.2. Clinical Endpoints and Follow-Up

The primary endpoint of the study was five-year all-cause mortality. Good clinical response, defined as an at least 15% increase of LVEF after six months of CRT, was considered as the secondary endpoint. All patients gave written informed consent before enrolment to the study. The investigation conformed to the Declaration of Helsinki; the study protocol was approved by the local Ethics Committee.

The follow-up period lasted five years; we conducted visits at six months, two years, and five years after CRT implantation. Detailed physical examinations, laboratory tests, ECG, and echocardiography were performed in a total of 136 patients at baseline. At follow-up visits, functional status of the patients was evaluated by assessing the NYHA classification; their medical therapy and relevant adverse medical events were documented. Laboratory blood analyses, echocardiography, and ECG were repeated at six months.

### 2.3. Laboratory Measurements, Exposure to Sunlight, and Echocardiography

We obtained venous blood samples from the patients, afterwards processed the serum and ethylenediaminetetraacetic acid plasma aliquots within two hours after sampling. Samples were stored at -80°C for later laboratory measurements. Total serum 25(OH)-vitamin D levels were measured with Roche Elecsys vitamin D total assay kits (Cat. No.: 05894913190, Roche Diagnostics, Mannheim, Germany. Reference value is >30 ng/mL in the Central Laboratory of Semmelweis University, respectively). N-terminal of the prohormone brain natriuretic peptide (NT-proBNP) levels was measured using Roche Elecsys NT-proBNP II kits (Cat. No.: 04842464190, Roche Diagnostics, Mannheim, Germany) with a Cobas e 411 analyzer (Roche Diagnostics, Mannheim, Germany). Serum calcium levels were measured CA2 kits (Cat. No.: 05061482190, Roche Diagnostics, Mannheim, Germany) with a Cobas Integra 400 Plus analyzer (Roche Diagnostics, Mannheim, Germany).

Exposure to sunlight was assessed by the cumulative hours of sunshine in the 30-day preceding enrolment, based upon the public databases of the National Meteorological Service [16].

Echocardiography and offline measurements were carried out by licensed echocardiographic experts using a Phillips iE 33 system, Philips Xcelera R3.1.L1, and Philips Qlab 9.0 software. LVEF was calculated using Simpson's biplane method. The reproducibility of echocardiographic measurements was determined; interobserver and intraobserver variability was assessed with Lin's concordance correlation coefficient using 12-12 pair of sample data; substantial correlation was proven, as described previously in this cohort (interobserver variability: *ρ*_c_ = 0.956 (0.89-0.98); intraobserver variability: *ρ*_c_ = 0.96 (0.89-0.97)).

### 2.4. Statistical Analysis

As the majority of the variables showed nonparametric distributions, the data were expressed as the medians with interquartile ranges or as percentages with the event numbers. Continuous variables were compared with the Wilcoxon matched pair test and the Mann-Whitney test, as appropriate. A chi-squared test was applied for categorical data comparisons. The Cox and univariate logistic regression analyses were applied to determine the baseline predictors of 5-year mortality and the lack of good clinical response; the continuous variables were standardized by one standard deviation increase for the regression analyses. We used receiver operating characteristic (ROC) analysis, and the continuous variables were dichotomized and then the Kaplan-Meier curves were compared using the log-rank tests. In the multivariable Cox regression and logistic regression models, the baseline model included variables with *p* < 0.1 value from the univariate analysis, and further adjusted models were built in a forward stepwise manner.

In the present study, a two-tailed *p* value of <0.05 was considered statistically significant. Statistical analyses were performed using IBM SPSS 22 (Apache Software Foundation, USA) and GraphPad Prism 6.03 (GraphPad Software, Inc., USA) software products.

## 3. Results

### 3.1. Baseline Characteristics and Effects of CRT on the Study Population

The baseline characteristics of the 136 patients are detailed in Table 1; the comparison of surviving patients and nonsurvivors is showed. Deceased patients had higher LVEF and NT-proBNP levels at baseline, while the proportion of patients with left bundle branch block (LBBB) and beta blocker therapy was significantly lower. Baseline plasma vitamin D level of the patients were 20.9 ng/mL (15.2-31.7), and we observed no significant change after six months of CRT (6 months: 21.5 (16.2-28.3), *p* = 0.43). The study population was described in detail previously; LVEF and left ventricular end-systolic and end-diastolic volumes (LVESV and LVEDV) decreased significantly [14].

### 3.2. Association of Baseline Vitamin D Concentrations with Clinical Outcomes

58 patients (43%) died during the 5-year follow-up; those who survived had significantly higher baseline vitamin D levels at baseline (23.07 (16.58-31.73) vs. 18.3 (13.81-23.75) ng/mL, *p* = 0.018). In 45 patients (33%), we observed the lack of good clinical response six months after CRT implantation; similarly, we measured increased baseline vitamin D levels in patients with good clinical response (22.56 (15.6-31.87) vs. 18.12 (13.95-23.43) ng/mL, *p* = 0.027).

**Figure 1:**
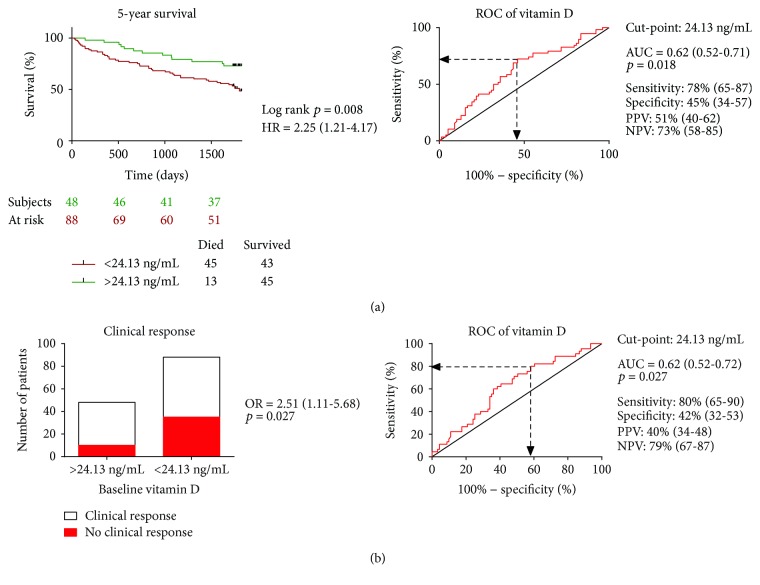
Impact of baseline vitamin D on five-year mortality and clinical response. Receiver operating characteristic analysis was performed for determining the optimal cutoff point for baseline plasma vitamin D levels. The odds and hazard ratios refer to the presence versus the absence of a baseline plasma vitamin D level < 24.13 ng/mL. (a) We compared the Kaplan-Meier survival curves by the log-rank test in patient groups of baseline plasma vitamin D levels below and over 24.13 ng/mL. We tested the 5-year mortality by using Cox regression analysis. (b) Clinical response, defined as a relative increase of at least 15% in the LVEF 6 months after implantation, was visualized by the contingency bar plot. We tested the lack of clinical response by using logistic regression analysis. HR: hazard ratio; OR: odds ratio; AUC: area under the curve; NPV: negative predictive value; PPV: positive predictive value.

To establish an optimal cut-point for the further assessment of the clinical outcomes, we used receiver operating characteristic analysis. Plasma vitamin D below 24.13 ng/mL seemed to be an optimal cut-point for 5-year mortality (AUC = 0.62 (0.52-0.71), *p* = 0.018; sensitivity: 78% (65-87); specificity: 45% (34-58)) and lack of 6-month clinical response (AUC = 0.62 (0.52-0.71), *p* = 0.027; sensitivity: 80% (65-90); specificity: 42% (32-53)), respectively (Figure 1).

**Figure 2:**
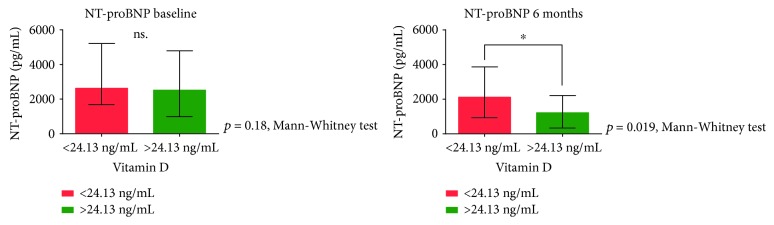
Plasma NT-proBNP levels at baseline and after six months of CRT. We compared the baseline and six-month plasma NT-proBNP levels in patients groups of baseline plasma vitamin D levels below and above 24.13 ng/mL using the Mann-Whitney test. CRT: cardiac resynchronization therapy; NT-proBNP: N-terminal of the prohormone brain natriuretic peptide.

**Figure 3:**
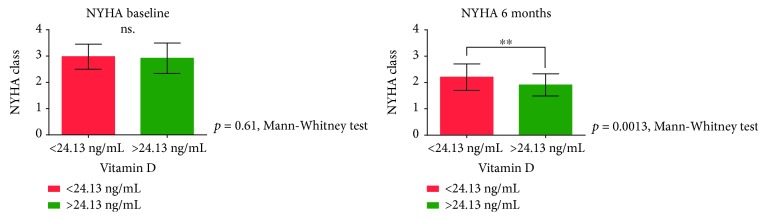
Severity of heart failure at baseline and after six months of CRT. We compared the baseline and six-month severity of heart failure using NYHA classification in patient groups of baseline plasma vitamin D levels below and above 24.13 ng/mL using the Mann-Whitney test. CRT: cardiac resynchronization therapy; NYHA class: classification of heart failure according to the New York Heart Association.

Next, we created groups of patients with baseline vitamin D below and over 24.13 ng/mL. The use of beta blockers was the only parameter that differed significantly between the subgroups; almost all patients in the group with higher vitamin D levels were on beta blockers (98% vs. 85%, *p* = 0.015). Sunlight exposure before enrolment did not differ significantly between groups (*p* = 0.56). When analyzing the severity of heart failure, there was no difference between groups at baseline reflected by the NT-proBNP levels (2626 (1683-5214) vs. 2518 (988-4791), *p* = 0.18) or the NYHA class (2.97 ± 0.48 vs. 2.91 ± 0.57, *p* = 0.61). NT-proBNP levels remained significantly elevated only in the subgroup of patients with low vitamin D levels (1216 (337-2214) vs. 2116 (927-3865) *p* = 0.019) (Figure 2). Moreover, their functional status (NYHA class) also did not improve (1.9 ± 0.42 vs. 2.21 ± 0.5, *p* = 0.001) (Figure 3).

### 3.3. Predictors of 5-Year Mortality and Lack of Good Clinical Response

We analyzed the 5-year all-cause mortality by univariate Cox regression and the six-month clinical response using univariate logistic regression analysis. Vitamin D level lower than 24.13 ng/mL was significantly associated with increased risk of long-term mortality (HR 2.25 (1.21-4.17), *p* = 0.008) and lack of good clinical response (OR 2.51 (1.11-5.68), *p* = 0.027) (Figure 1).

As described in detail previously, LBBB (*p* < 0.0001), use of beta blocker therapy (*p* = 0.003), and increasing NT-proBNP levels (*p* < 0.0001) predicted all-cause mortality; increasing age proved to bear a marginally significant predictive capacity (*p* = 0.07). Relevant baseline clinical variables related to lack of good clinical response were hypertension (*p* = 0.08), hyperlipidaemia (*p* = 0.09), mineralocorticoid receptor inhibitor therapy (*p* = 0.06), and increasing levels of NT-proBNP (*p* = 0.22). The detailed results of univariate logistic and Cox regression statistical analyses are showed in Supplementary [Supplementary-material supplementary-material-1].

To determine the independent influence of decreased vitamin D levels on mortality, we set up a basic multivariable Cox regression model with the baseline clinical variables shown to be relevant by the univariate analysis (*p* < 0.10). Thus, the baseline multivariable model included age, LBBB, use of beta blocker therapy, and baseline NT-proBNP. In the following step, we entered vitamin D levels into the baseline model in a forward stepwise way. Vitamin D levels under 24.13 ng/mL predicted mortality in the multivariable model as well (HR = 1.92 (1.02-1.45), *p* = 0.045).

We use the same method to investigate the clinical response. We included the relevant factors to the basic multivariable model: hypertension, hyperlipidemia, mineralocorticoid receptor inhibitor therapy, and increasing levels of NT-proBNP. We entered vitamin D levels into the baseline model in a forward stepwise way. Similar to mortality prediction, vitamin D was an independent predictor of lack of good clinical response (OR = 2.62 (1.01-6.25), *p* = 0.03). The detailed results of multivariate logistic and Cox regression statistical analyses are showed in Supplementary [Supplementary-material supplementary-material-1].

## 4. Discussion

### 4.1. Synopsis of Key Findings

Vitamin D levels under 24.13 ng/mL predicted long-term mortality and poor clinical response in HF patients undergoing CRT independently of all relevant baseline predictors including NT-proBNP. Furthermore, patients with vitamin D insufficiency had significantly higher NT-proBNP levels and suffered from more severe HF six months after CRT.

### 4.2. Possible Mechanisms and Explanation

Chronic HF is an emerging disease in the high- and middle-income countries, affecting millions of people and requiring considerable amount of healthcare expenditure. HF development is considered a compound pathophysiological process, involving the activation of neurohormonal and inflammatory pathways, tissue remodeling. Wide range of pharmaceutical and device-based treatment is available, yet the overall outcome of HF is still poor [17].

Vitamin D has been considered the key regulator of calcium and phosphorus homeostasis and bone mineralization. Besides the regulation of bone metabolism, recent studies showed that vitamin D has numerous extraskeletal functions. It plays various regulatory roles in several mechanisms considered fundamental in development of HF [18]. Vitamin D suppresses the expression of renin and RAAS activity [19] and modulates the turnover of the ECM by enhancing the production of matrix metalloproteinase inhibitors [20], and experimental studies showed that it promotes myocyte contraction and relaxation by modulating the calcium influx [21]. In case of vitamin D deficiency, with the loss of the previous regulating effects, hypertrophy, ECM deposition, and myocardial fibrosis may arise.

Clinical studies also confirmed that vitamin D has a strong impact in HF. The risk of developing HF was increased in longitudinal studies in case of low levels of vitamin D, while prevalent vitamin D deficiency was found among HF patients [6]. Furthermore, it was associated with significantly worse prognosis [22, 23].

Although vitamin D deficiency contributes to the development of HF by several described regulatory mechanisms, the clear beneficial effect of vitamin D supplementation in HF patients is still under debate, based upon recent randomized studies [24, 25]. Further randomized studies with consistent enrolment criteria are needed to validate the benefit.

Previous small studies also suggested that low levels of vitamin D predict poor 6-month response after CRT implantation [8, 9], yet there was no data available regarding long-term mortality. In our study, we also confirmed the role of vitamin D in the prediction of the lack of good clinical response after CRT. Baseline vitamin D levels under 24.13 ng/mL were significantly associated with poor clinical response, independently of all relevant baseline predictors. Patients with lower baseline vitamin D levels had 2.5-fold risk of lack of good clinical response after CRT.

Furthermore, we demonstrated that reduced baseline vitamin D levels were significant predictors of 5-year mortality after CRT; the mortality risk was independent of all relevant baseline predictors. Patients with vitamin D levels under 24.13 ng/mL had more than 2-fold mortality risk during the follow-up.

We used the NYHA classification of HF and NT-proBNP levels as surrogate markers to further assess the clinical response of patients. Interestingly, at baseline there were no significant differences between groups of patients with vitamin D levels below and above 24.13 ng/mL. After six months of CRT, patients with reduced vitamin D levels had significantly higher NT-proBNP levels and NYHA classes, indicating a more severe stage of HF, enhanced progression, and poor clinical response.

According to the 2011 classification by the Institute of Medicine, 25(OH)-vitamin D levels below 20 ng/mL are considered inadequacy, while levels between 20 and 50 ng/mL are considered adequacy [26]. Our statistically verified cut-point reaching almost the limit of vitamin D inadequacy also supports the previous findings regarding the close association of low vitamin D levels and poor clinical outcomes in HF.

### 4.3. Limitations

Remarkable limitations of our study are the relatively small sample size and the single-center design. All-cause mortality was considered as the primary endpoint; cause of death was not investigated separately in this analysis due to the relatively low event numbers. The assessment of the sunlight exposure of patients can only credited as an approximate, since the average cumulative hours of sunshine in Hungary were calculated. Our results can be represented as hypothesis-generating findings; this observation does not prove direct causality between vitamin D levels and prognosis in HF patients undergoing CRT. Validation with prospective, multicenter studies is necessary.

## 5. Conclusion

Our results further verify the previous findings concerning the association of reduced vitamin D levels with the poor prognosis in various HF populations. Decreased baseline vitamin D levels predicted poor outcomes after CRT, functional status, and clinical response. Long-term survival of the patients was significantly worse compared with patients with adequate baseline vitamin D levels. Therefore, assessing the vitamin D homeostasis of HF patients before CRT implantation might be helpful in identifying the high-risk patients, among whom nonresponse should be anticipated.

## Figures and Tables

**Table 1:** Baseline characteristics.

Clinical variables	All patients (*n* = 136)	Surviving patients (*n* = 78)	Deceased patients (*n* = 58)	*p* value
Age (years)	67 (60-73)	67 (60-71)	70 (62-74)	0.067
Gender (male)	81 (110)	76 (59)	88 (51)	0.078
BMI (kg/m^2^)	27 (24-30)	27 (25-30)	27 (23-29)	0.196
Ischemic HF	57 (78)	53 (41)	64 (37)	0.201
LBBB	82 (112)	91 (71)	71 (41)	0.003
CRT-D	16 (22)	18 (14)	14 (8)	0.533
Opt. lead position	74 (100)	73 (57)	74 (43)	0.896
QRS (msec)	163 (141-184)	164 (141-184)	163 (144-185)	0.691
LVEF (%)	28 (23-33)	28 (23-32)	34 (25-40)	<0.001
LVESV (mL)	211 (154-276)	218 (160-276)	207 (141-268)	0.353
LVEDV (mL)	303 (251-361)	313 (251-382)	299 (242-343)	0.448
NYHA III- IV	86 (117)	86 (117)	91 (53)	0.138
Hypertension	56 (76)	55 (43)	57 (33)	0.054
Hyperlipidemia	24 (33)	22 (17)	28 (16)	0.423
Diabetes mellitus	37 (50)	33 (26)	41 (24)	0.339
ACEi/ARB	96 (130)	97 (76)	93 (54)	0.277
BB	90 (122)	95 (74)	83 (48)	0.022
MRI	71 (96)	74 (58)	65 (33)	0.258
Calcium (mmol/L)	2.43 (2.34-2.49)	2.43 (2.36-2.49)	2.41 (2.32-2.50)	0.922
NT-proBNP (pg/mL)	2612 (1377-5124)	2101 (1000-3555)	4035 (2125-6479)	<0.001
Sunlight (hours)	156 (67-241)	157 (102-241)	148 (66.7-221)	0.748

Data is expressed as median with interquartile range for continuous variables and as percentage with event numbers for categorical variables. BMI: body mass index; ischemic HF: ischemic etiology of the heart failure; LBBB: left bundle branch block; CRT-D: cardiac resynchronization therapy with implantable cardioverter defibrillator; Opt. lead position: lateral or posterolateral left ventricular lead position; LVEF: left ventricular ejection fraction; LVESV: left ventricular end systolic volume; LVEDV: left ventricular end diastolic volume; NYHA III-IV: New York Heart Association classification 3-4; ACEi/ARB: angiotensin convertase inhibitor/angiotensin receptor blocker therapy; BB: beta blocker therapy; MRI: mineralocorticoid receptor inhibitor therapy; NT-proBNP: N-terminal of the prohormone brain natriuretic peptide, sunlight: cumulative duration of sunlight in the 30 days prior enrolment.

## Data Availability

The data used to support the findings of this study are available from the corresponding author upon request.

## Supplementary Materials

Supplementary Materials Supplementary Tables 1 and 2: the detailed results of logistic and Cox regression statistical analyses are presented, including both univariate and multivariate models.
