# Supplementary material for: Vitamin D Deficiency Predicts Poor Clinical Outcomes in Heart Failure Patients Undergoing Cardiac Resynchronization Therapy
Source: Dis Markers. 2019 Oct 13;2019:4145821. doi: 10.1155/2019/4145821 (PMC6815629; doi:10.1155/2019/4145821)
Supplement: Supplementary Materials — Supplementary Tables 1 and 2: the detailed results of logistic and Cox regression statistical analyses are presented, including both univariate and multivariate models. [file 4145821.f1.docx]

| Suplementary Table 1. Baseline parameters as predictors of the good clinical response and the 5-year mortality | | | | | | | | | |
| --- | --- | --- | --- | --- | --- | --- | --- | --- | --- |
| **Heart failure patients (n=136)** | **Lack of good clinical response (n=45)** | | | |  | **5-year mortality (n=58 )** | | | |
|  | **OR** | **95% CI of OR** | **Wald χ^2^** | **p** |  | **HR** | **95% CI of HR** | **Wald χ^2^** | **p** |
| **Clinical variables** |  |  |  |  |  |  |  |  |  |
| Age (years) | 1.27 | 0.87-1.85 | 1.46 | 0.23 |  | 1,30 | 0,97 - 1,73 | 3,25 | 0,07 |
| Male gender | 1.83 | 0.68-4.94 | 1.43 | 0.23 |  | 1,87 | 0,85 - 4,14 | 2,44 | 0,11 |
| BMI (kg/m^2^) | 1.18 | 0.83-1.69 | 0.83 | 0.36 |  | 0,85 | 0,65 - 1,11 | 1,40 | 0,23 |
| Ischaemic HF | 0.97 | 0.47-2.01 | 0.01 | 0.94 |  | 1,42 | 0,83 - 2,43 | 1,68 | 0,19 |
| LBBB | 1.60 | 0.59-4.37 | 0.85 | 0.36 |  | 0,36 | 0,20 -0,63 | 12,25 | <0,0001 |
| CRT-D | 1.19 | 0.46-3.08 | 0.13 | 0.72 |  | 0,72 | 0,34 - 1,53 | 0,70 | 0,40 |
| Opt. Lead position | 1.17 | 0.52-2.66 | 0.14 | 0.71 |  | 1,00 | 0,55 - 1,81 | 0,00 | 0,98 |
| QRS (msec) | 0.81 | 0.56-1.17 | 1.28 | 0.26 |  | 0,95 | 0,73 - 1,23 | 0,12 | 0,72 |
| EF (%) | 3.37 | 2.01-5.66 | 21.0 | >0.001 |  | 0,97 | 0,74 - 1,27 | 0,03 | 0,86 |
| ESV (ml) | 0.71 | 0.48-1.05 | 2.91 | 0.09 |  | 0,92 | 0,70 - 1,21 | 0,32 | 0,57 |
| EDV (ml) | 0.71 | 0.48-1.05 | 3.00 | 0.08 |  | 0,92 | 0,71 -1,21 | 0,30 | 0,57 |
| NYHA III,IV | 0.83 | 0.30-2.26 | 0.14 | 0.71 |  | 2,07 | 0,83 - 5,20 | 2,44 | 0,11 |
| Hypertension | 1.96 | 0.93-4.12 | 3.13 | 0.08 |  | 1,05 | 0,62 - 1,76 | 0,03 | 0,85 |
| Hyperlipidaemia | 2.02 | 0.91-4.54 | 2.95 | 0.09 |  | 1,13 | 0,63 - 2,01 | 0,17 | 0,67 |
| Diabetes mellitus | 0.93 | 0.44-1.95 | 0.04 | 0.84 |  | 1,49 | 0,88 -2,51 | 2,28 | 0,13 |
| ACEi/ARB | 0.98 | 0.17-5.61 | 0.01 | 0.99 |  | 0,54 | 0,19 - 1,50 | 1,38 | 0,24 |
| BB | 0.63 | 0.20-1.93 | 0.66 | 0.42 |  | 0,35 | 0,17 - 0,69 | 8,98 | 0,003 |
| MRI | 0.48 | 0.22-1.03 | 3.57 | 0.06 |  | 0,72 | 0,42 - 1,24 | 1,39 | 0,23 |
| NT-proBNP (pg/ml) | 1.24 | 0.88-1.75 | 1.52 | 0.22 |  | 1,43 | 1,19 - 1,72 | 15,03 | **<**0,0001 |
| Vitamine D < 24.13 ng/ml | 2.51 | 1.11-5.68 | 4.87 | 0.027 |  | 2.25 | 1.21-4.17 | 6.64 | 0.008 |
| Data is expressed as median with interquartile range for continuous variables and as event numbers with percentage for categorical variables. Lack of good clinical response (≥ 15 % increase in the LVEF) was tested by using the univariate logistic regression analysis and the 5-year mortality was assessed by using the univariate Cox regression analysis. The continuous variables were standardized by 1 standard deviation increase. The odds and hazard ratios refer for presence versus absence in case of categorical variables and 1 standard deviation increase in case of continuous variables. OR = odds ratio; HR= hazard ratio; CI = confidence interval; χ2 = Chi squared; BMI = body mass index; Ischaemic = ischaemic etiology of the heart failure; LBBB = left bundle branch block; CRT-D = cardiac resynchronization therapy with implantable cardioverter defibrillator; Opt. lead position = lateral or postero-lateral left ventricular lead position; LVEF = left ventricular ejection Fraction; LVESV = left ventricular end systolic volume; LVEDV = left ventricular end diastolic voulme; NYHA III,IV = New York Heart Association classification 3-4; ACEi/ARB = angiotensin convertase inhibitor /angiotensin receptor blocker; BB = beta blocker; MRI = mineralocorticoid receptor inhibitor; NT-proBNP = N-terminal of the prohormone brain natriuretic peptide. | | | | | | | | | |
|  |  |  |  |  |  |  |  |  |  |
|  |  |  |  |  |  |  |  |  |  |
|  |  |  |  |  |  |  |  |  |  |
|  |  |  |  |  |  |  |  |  |  |
|  |  |  |  |  |  |  |  |  |  |
|  |  |  |  |  |  |  |  |  |  |
|  |  |  |  |  |  |  |  |  |  |
|  |  |  |  |  |  |  |  |  |  |

| Supplementary Table 2. Multivariable prediction models, including all significant baseline parameters in the baseline model | | | | | | | | | |
| --- | --- | --- | --- | --- | --- | --- | --- | --- | --- |
|  | **Lack of 6-month clinical response (n=45)** | | | | **5-year mortality (n=58 )** | | | | |
|  | **OR** | **95% CI** | **Wald** | **p value** | **HR** | **95% CI** | **Wald** | **p value** |  |
| Vitamin D < 24.13 ng/mL | 2.62 | 1.01-6.25 | 4.72 | 0.03 | 1.92 | 1.02-1.45 | 2.62 | 0.045 |  |
| The lack of 6-month clinical response (n=45) was defined as at least 15 % increase in the left ventricular ejection fraction. The baseline model for the multivariable logistic regression analysis included hypertension, hyperlipidaemia, mineralocorticoid receptor inhibitor therapy, and increasing levels of NT-proBNP. The baseline model for the 5-year mortality (n=58) using multivariable Cox regression analysis included age, left bundle branch block, beta blocker therapy, and NT-proBNP. In a forward stepwise way we adjusted Vitamin D levels under 24.13 ng/mL to the basic models. The odds and hazard ratios refer for Vitamin D levels under 24.13 ng/mL. OR = odds ratio; HR= hazard ratio; CI = confidence interval; χ2 = Chi squared; NT-proBNP = N-terminal of the prohormone brain natriuretic peptide. | | | | | | | | | |
|  |  |  |  |  |  |  |  |  |  |
|  |  |  |  |  |  |  |  |  |  |
|  |  |  |  |  |  |  |  |  |  |
|  |  |  |  |  |  |  |  |  |  |
